# β-Hydroxybutyrate upregulates FGF21 expression through inhibition of histone deacetylases in hepatocytes

**DOI:** 10.1515/biol-2022-0095

**Published:** 2022-08-10

**Authors:** Aili Yan, Yanyan Zhao, Lijun Zhang, Xiangyan Liang, Xiaochun Zhang, Fenli Liang, Shen Nian, Xinhua Li, Zhuo Sun, Ke Li, Yu-Feng Zhao

**Affiliations:** Institute of Basic Medical Sciences, Xi’an Medical University, Xi’an, 710021, China; Institute of Basic and Translational Medicine, Xi’an Medical University, Xi’an, 710021, China

**Keywords:** β-hydroxybutyrate, fibroblast growth factor 21, hepatocyte, histone deacetylases

## Abstract

Fibroblast growth factor 21 (FGF21) is secreted by hepatocytes as a peptide hormone to regulate glucose and lipid metabolism. FGF21 promotes hepatic ketogenesis and increases ketone body utilization in starvation. Histones are the target molecules of nutrients in regulating hepatic metabolic homeostasis. However, the effect of ketone bodies on FGF21 expression and the involvement of histones in it is not clear yet. The present study observed the effects of β-hydroxybutyrate (β-OHB), the main physiological ketone body, on FGF21 expression in human hepatoma HepG2 cells *in vitro* and in mice *in vivo*, and the role of histone deacetylases (HDACs) in β-OHB-regulated FGF21 expression was investigated. The results showed that β-OHB significantly upregulated FGF21 gene expression and increased FGF21 protein levels while it inhibited HDACs’ activity in HepG2 cells. HDACs’ inhibition by entinostat upregulated FGF21 expression and eliminated β-OHB-stimulated FGF21 expression in HepG2 cells. Intraperitoneal injections of β-OHB in mice resulted in the elevation of serum β-OHB and the inhibition of hepatic HDACs’ activity. Meanwhile, hepatic FGF21 expression and serum FGF21 levels were significantly increased in β-OHB-treated mice compared with the control. It is suggested that β-OHB upregulates FGF21 expression through inhibition of HDACs’ activity in hepatocytes.

## 1 Introduction

Neuro-endocrine signals finely control body fuel metabolism in response to nutritional status. In starvation, fibroblast growth factor 21 (FGF21) is primarily expressed and secreted by hepatocytes, and it acts as a protein hormone to regulate glucose and lipid metabolism for the extension of survival [1]. FGF21 has broad metabolic actions in rodents and primates. It enhances glucose utilization, decreases blood triglyceride concentration, promotes weight loss, and reduces inflammation in obese subjects [2,3,4,5]. Thus, FGF21 has been a promising drug candidate for treating metabolic diseases, such as type 2 diabetes, obesity, and nonalcoholic steatohepatitis.

Upregulation of hepatic FGF21 expression may represent a way to treat metabolic diseases [6]. FGF21 expression is regulated by hormones, nutrients, and cell stress-inducing factors, including glucagon, thyroid hormone, lipoic acid, retinoic acid, and curcumin [7,8,9,10]. Multiple intracellular signaling molecules, such as peroxisome proliferator activator receptor-α (PPAR-α), AMP-activated kinase (AMPK), and activating transcriptional factors, have been demonstrated to regulate hepatic FGF21 expression [11,12]. New regulators of hepatic FGF21 expression and their regulatory mechanism are expected to enrich this field of research.

Epigenetics plays an important role in regulating gene expression, and histone acetylation and deacetylation are among the principal mechanisms of epigenetic gene expression [13]. Histone deacetylases (HDACs) are a group of enzymes that can remove the acetyl group on the lysine of histone protein, thereby affecting the gene transcription [14]. HDAC inhibitors, such as entinostat, have become candidate drugs for tumor treatment by affecting cell gene expression [15,16]. HDACs may also be involved in hepatic gene expression, including FGF21 expression [17].

In starvation, the hepatocyte produces ketone bodies for energy supply to extrahepatic organs, such as the brain and heart [18]. β-Hydroxybutyrate (β-OHB) is the major component of ketone bodies, and it elevates from less than 0.1 mM with a normal diet to more than 4 mM under a ketogenic diet and in starvation [19,20]. β-OHB has diverse physiological functions and displays therapeutic effects on tumors and neurologic disorders [21], and some reports showed that β-OHB inhibits HDACs’ activities [22,23,24]. However, the role of ketone bodies in FGF21 expression is not clear yet. The present study observed the effect of β-OHB on hepatic FGF21 expression and secretion and investigated its underlying mechanism.

## 2 Materials and methods

### 2.1 Materials

β-OHB and 3-(4,5-dimethylthiazol-2-yl)-2,5-diphenyltetrazolium bromide (MTT) were purchased from Sigma-Aldrich (Merck KGaA, Darmstadt, Germany). Entinostat was obtained from Selleck Chemicals (Houston, TX, USA). Dulbecco’s modified eagle medium (DMEM), fetal bovine serum (FBS), and the other reagents for cell culture were the products of Thermo Fisher (Waltham, MA, USA). The kits for RNA extraction, reverse transcription (RT), quantitative PCR (qPCR) and the kits for cellular protein extraction and measurement were obtained from Takara Bio USA Inc (Mountain View, CA, USA). HDAC activity colorimetric assay kits were the products of BioVision Incorporated (Milpitas, CA, USA). Histone H3ac (pan-acetyl) polyclonal antibody, glyceraldehyde phosphate dehydrogenase (GAPDH) polyclonal antibody, horseradish peroxidase (HRP)-conjugated goat-anti rabbit antibody, and 20× Tris-buffered solution (TBS) were purchased from Thermo Fisher. Human and mouse FGF21 ELISA kits, glutamic–pyruvic transaminase (ALT) activity assay kit, and glutamic–oxaloacetic transaminase (AST) activity assay kit were the products of Elabscience (Wuhan, China). Hematoxylin–eosin (H&E) staining kits were obtained from Beyotime Biotechnology (Beijing, China).

### 2.2 Cell culture

Human hepatoma HepG2 cell was obtained from American Type Culture Collection (ATCC, Washington, DC, USA) and cultured in DMEM containing 5.6 mM glucose and 10% FBS. The cells were refreshed by DMEM without FBS before they were treated by β-OHB and entinostat (5 μM). Experiments with increasing concentration of β-OHB were performed (1, 2, 4, 8 mM).

### 2.3 RT-qPCR

Total RNA extraction and RT were performed using the kits, and FGF21 gene expression was measured by qPCR with SYBR premix EX Taq. The comparative Ct (cycle threshold) values were normalized to GAPDH, and 2^−ΔΔCt^ method was used to calculate the relative levels of gene expression. The primers were listed as follows: human FGF21 forward 5′-GGG AGT CAA GAC ATC CAG GT-3′ and reverse 5′-GGC TTC GGA CTG GTA AAC AT-3′; human GAPDH forward 5′-GAC AAC TTT GGT ATC GTG G-3′ and reverse 5′-AGG CAG GGA TGA TGT TCT-3′; mouse FGF21 forward 5′-TCC AAA TCC TGG GTG TCA AA-3′ and reverse 5′-CAG CAG CAG TTC TCT GAA GC-3′; and mouse GAPDH forward 5′-GAG AAC TTT GGC ATT GTG G-3′ and reverse 5′-ATG CAG GGA TGA TGT TCT G-3′.

### 2.4 ELISA

FGF21 levels in culture medium and in mouse serum were measured by ELISA kits according to the instruction. Briefly, the samples and the standards were added to 96-well plates in duplicate for 1.5 h incubation at 37°C, and then, the biotinylated antibodies were added for 1 h incubation at 37°C. After being washed with phosphate-buffered saline three times, the plates were incubated with avidin–HRP conjugates for 30 min at 37°C. Then, the plates were washed five times, and the substrate reagents were added for color development. After the color development was stopped, the optical density (OD) value per well was measured at 450 nm, and FGF21 levels in samples were calculated according to the standard curve.

### 2.5 Cell viability assay

After growing up to 60% confluence in 96-well plates, HepG2 cells were changed to serum-free medium and treated by β-OHB for 12 h, and then, MTT was added into medium (0.5 mg/ml in final concentration) for 4 h incubation. MTT crystals were dissolved by acidic isopropanol, and OD values at 560 nm were measured for statistical analysis.

### 2.6 HDACs’ activity assay

The homogenates of HepG2 cells and mouse liver were prepared, and the HDACs’ activity was measured using the kits according to the instruction. First, the homogenates (25 μg/sample) were mixed with HDAC assay buffer and HDAC colorimetric substrate for 90 min incubation at 37°C, which was followed by the addition of a lysine developer. Then, the OD values at 405 nm were read to represent HDACs’ activity.

### 2.7 Western blot

The total proteins of HepG2 cells were extracted and prepared for electrophoresis and then transferred to a nitrocellulose membrane. The membranes were incubated in sequences by blocking buffer for 2 h at room temperature, rabbit-anti H3ac antibody (1:1,000) or rabbit-anti GAPDH antibody (1:1,000) overnight at 4°C, HRP-conjugated goat anti-rabbit IgG antibodies for 1 h incubation at 37°C and chemiluminescent substrates, with TBS washing between each two steps. The luminescence was imaged by the ChemiDoc MP gel imaging analysis system.

### 2.8 Animal experiment

The 8-week-old male C57BL/6J mice were housed in the condition of a 12-h light/dark cycle, 22 ± 1°C and 55% humidity, with free access to food and water. They were divided into the β-OHB group and the control group (8 mice per group). After the blood samples (100 μl/mouse) were collected via the tail vein, the mice were given β-OHB via intraperitoneal injections four times with 2-h intervals in the β-OHB group (0.5 g/kg body weight per time), while the control mice were injected with saline in the same way. Finally, the mice were anesthetized by isoflurane inhalation and decapitated to collect serums and liver tissues. Liver tissues were used for the observation of gene expression and HDACs’ activity, and serums were used for measuring FGF21 and β-OHB levels as well as AST and ALT activities.


**Ethical approval:** The research related to animal use has been complied with all the relevant national regulations and institutional policies for the care and use of animals. The mice in this study followed the regulation of Assessment and Accreditation of Laboratory Animal Care. The animal study protocol was approved by the Ethics Committee of Xi’an Medical University (2015KTCQ03-03).

### 2.9 ALT and AST assays

ALT and AST activities in mouse serum were measured using the colorimetric assay kits according to the instruction. Briefly, the serum samples were mixed with buffer solution and substrate buffer for 30 min at 37°C. The chromogenic agent was then added for 20 min incubation at 37°C. Finally, a stop solution was added, and the OD values at 510 nm were read for the calculation of AST and ALT activities according to the standard curve.

### 2.10 H&E staining

The liver tissues were fixed with 4% paraformaldehyde and then routinely embedded with paraffin to prepare slides. H&E staining was carried out in slides according to the instruction, and the images were photographed.

### 2.11 Statistical analysis

Data were expressed as means ± SEM. D’Agostino-Pearson omnibus test was applied to test data distribution normality. Comparisons of means of multiple groups were analyzed via one-way analysis of variance followed by Bonferroni *post hoc* tests for normally distributed data. Abnormally distributed data were analyzed via the Kruskal–Wallis *H* test. *P* < 0.05 was considered to be a statistically significant difference.

## 3 Results

### 3.1 β-OHB stimulated FGF21 expression and secretion in HepG2 cells

**Figure 1:**
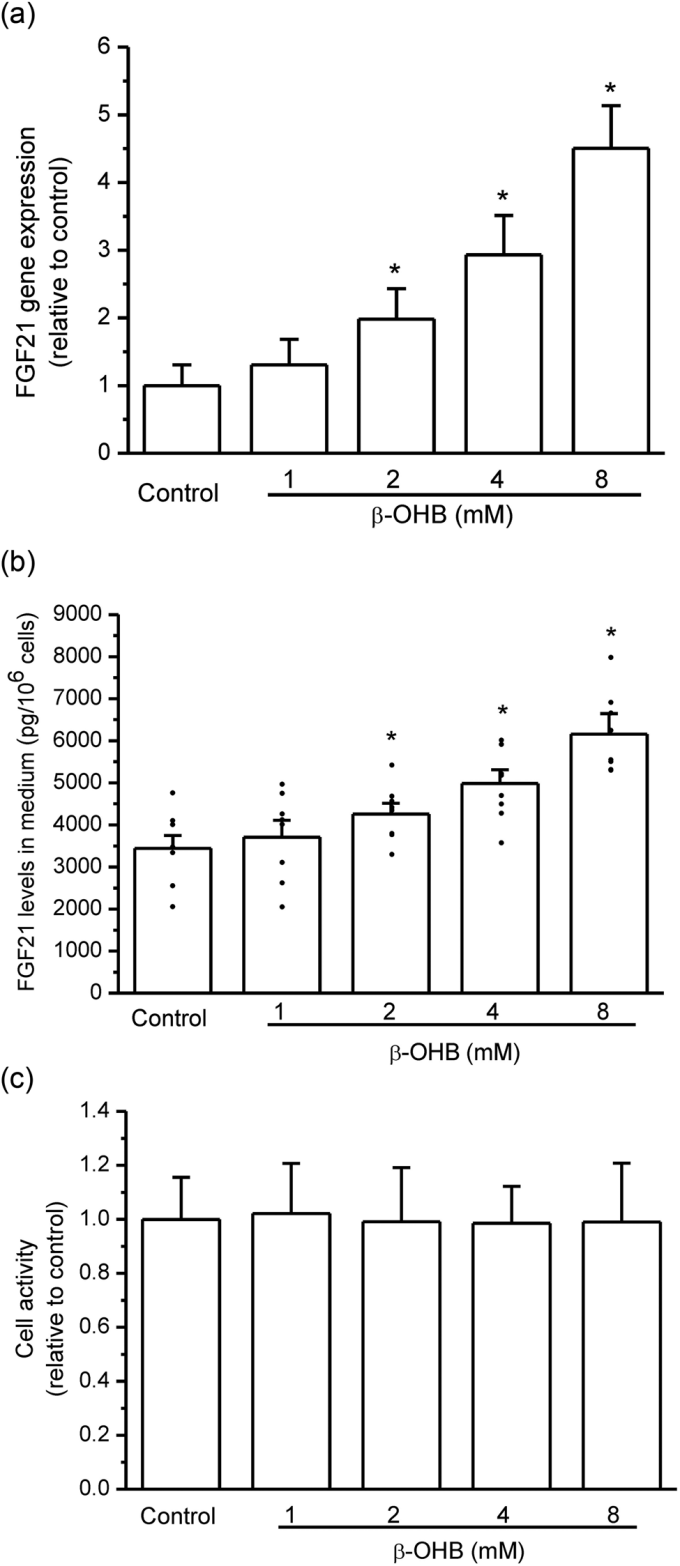
β-OHB stimulated FGF21 expression and secretion in HepG2 cells. (a) β-OHB significantly upregulated FGF21 gene expression after 12 h treatment in HepG2 cells (*P < 0.05 vs control, *n* = 3 times repetitions); (b) FGF21 protein levels in cell medium significantly increased after β-OHB treatment (*P < 0.05 vs control, *n* = 8 samples per group); and (c) the cell viability was not different between the two groups (no significance vs control, *n* = 8 samples per group).

As shown by RT-qPCR, β-OHB dose-dependently (1–8 mM) upregulated FGF21 gene expression after 12 h treatment in HepG2 cells. β-OHB displayed a significant effect at 2 mM and stronger effects with the increase in doses (*P* < 0.05, *n* = 3, Figure 1a). Accordant to FGF21 gene expression, FGF21 protein levels in the cell medium also significantly increased after β-OHB treatment (*P* < 0.05, *n* = 8, Figure 1b). The cell viability was not significantly influenced by β-OHB in 12 h treatment within 8 mM as shown by the MTT assay (Figure 1c).

### 3.2 β-OHB inhibited HDACs’ activity in HepG2 cells

**Figure 2:**
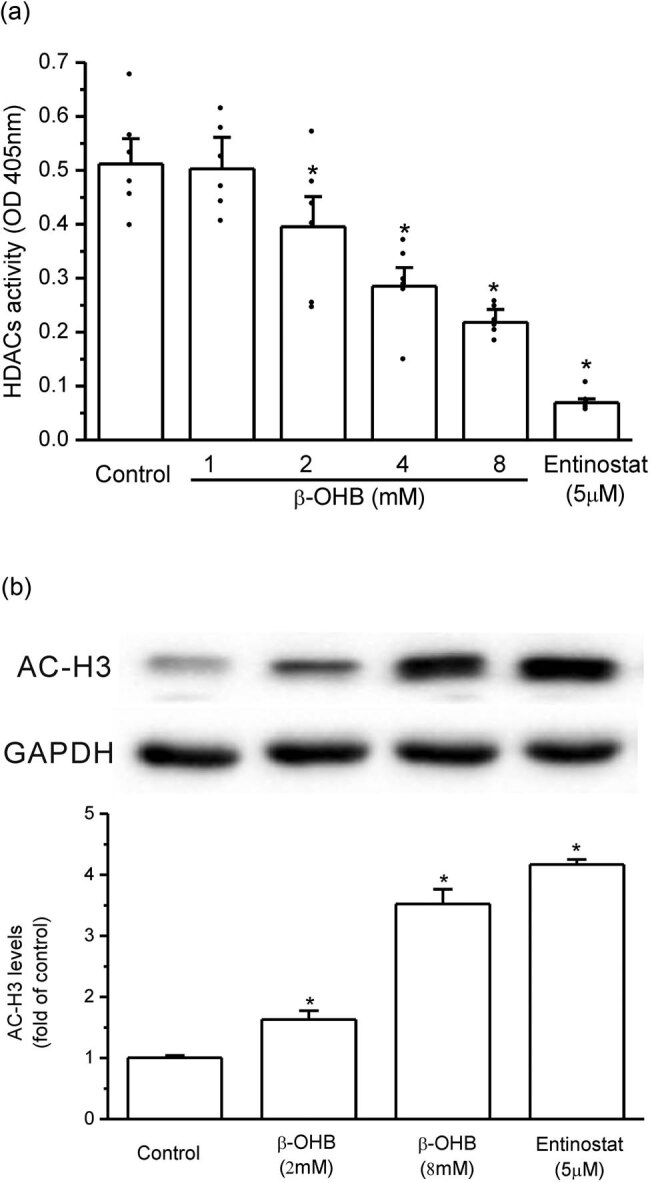
β-OHB inhibited HDACs’ activity in HepG2 cells. (a) β-OHB dose-dependently inhibited HDACs’ activity in HepG2 cells (*P < 0.05 vs control, *n* = 6 samples per group). (b) The levels of AC-H3 in HepG2 cells were significantly increased by β-OHB treatment as shown by western blot (*P < 0.05 vs control, *n* = 3 times repetitions).

As shown in Figure 2a, β-OHB significantly inhibited HDACs’ activity in HepG2 cells, although its effect was less than that of entinostat, the class I HDAC inhibitor (*P* < 0.05, *n* = 6). Accordant to the inhibition of HDACs by β-OHB, the levels of acetylated histone H3 (AC-H3) in HepG2 cells were significantly increased by β-OHB and entinostat as shown by western blot (*P* < 0.05, *n* = 3, Figure 2b).

### 3.3 HDACs’ inhibition mediated β-OHB-stimulated hepatic FGF21 expression

**Figure 3:**
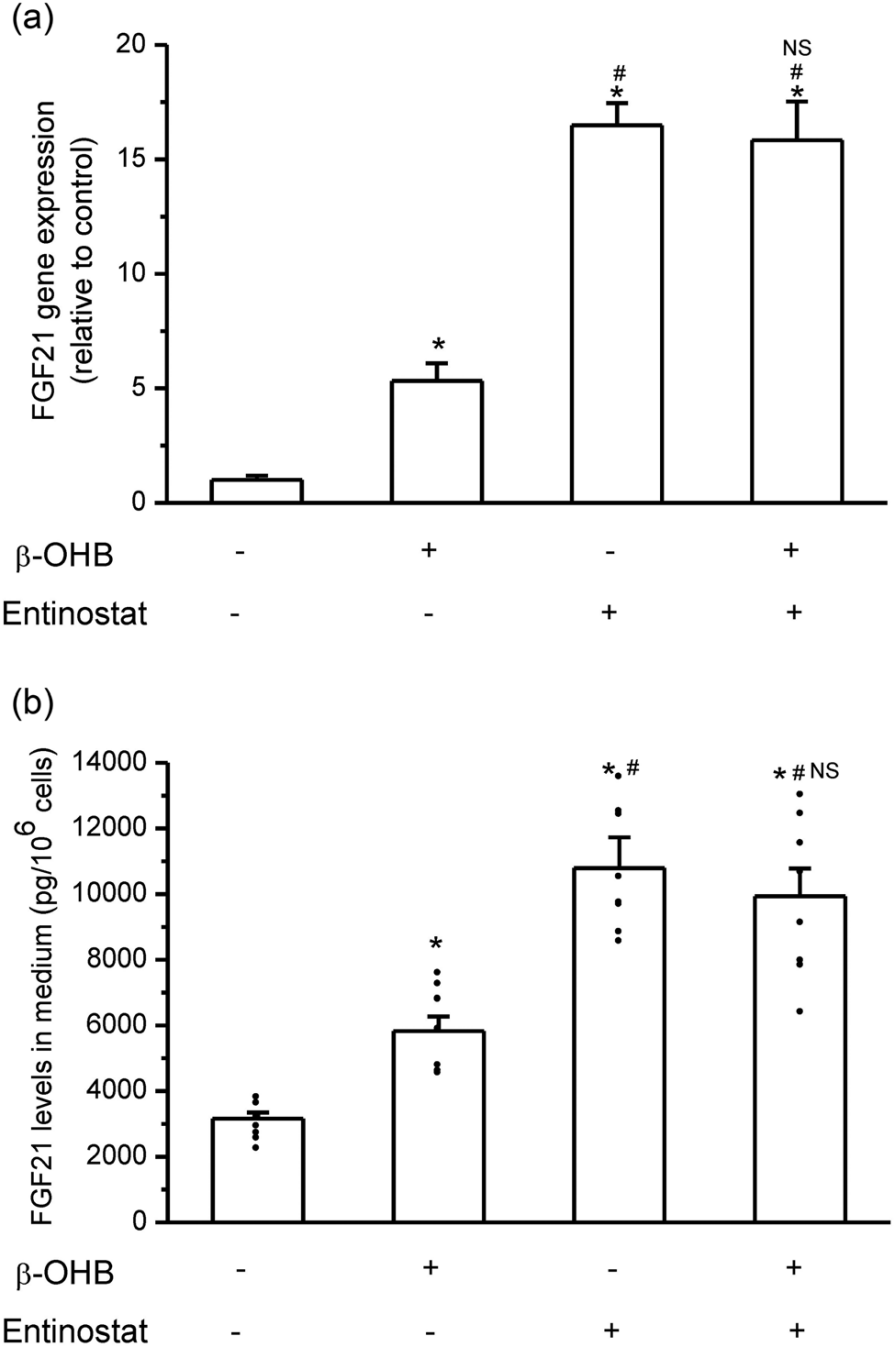
HDAC inhibition was involved in β-OHB-stimulated FGF21 expression and secretion in HepG2 cells. (a) HDACs’ inhibitor entinostat stimulated FGF21 gene expression, and there was no superposition effect of β-OHB on FGF21 expression on the basis of entinostat (*P < 0.05 vs control, #*P* < 0.05 vs single β-OHB group, NS means no significance vs single entinostat group, *n* = 3 times repetitions). (b) Entinostat increased FGF21 protein levels in medium, and no superposition effect of β-OHB on FGF21 protein levels was observed (*P < 0.05 vs control, #*P* < 0.05 vs single β-OHB group, NS means no significance vs single entinostat group, *n* = 8 samples per group).

The effects of HDACs’ inhibition on β-OHB-stimulated FGF21 expression in HepG2 cells were then observed. Entinostat (5 μM) significantly increased FGF21 expression and secretion in HepG2 cells. In combination with entinostat (5 μM), the stimulatory effect of β-OHB (8 mM) on FGF21 expression and secretion was fully eliminated, and no superposition effect of β-OHB based on entinostat was observed (Figure 3a and b).

### 3.4 β-OHB stimulated FGF21 expression in mouse liver *in vivo*

**Figure 4:**
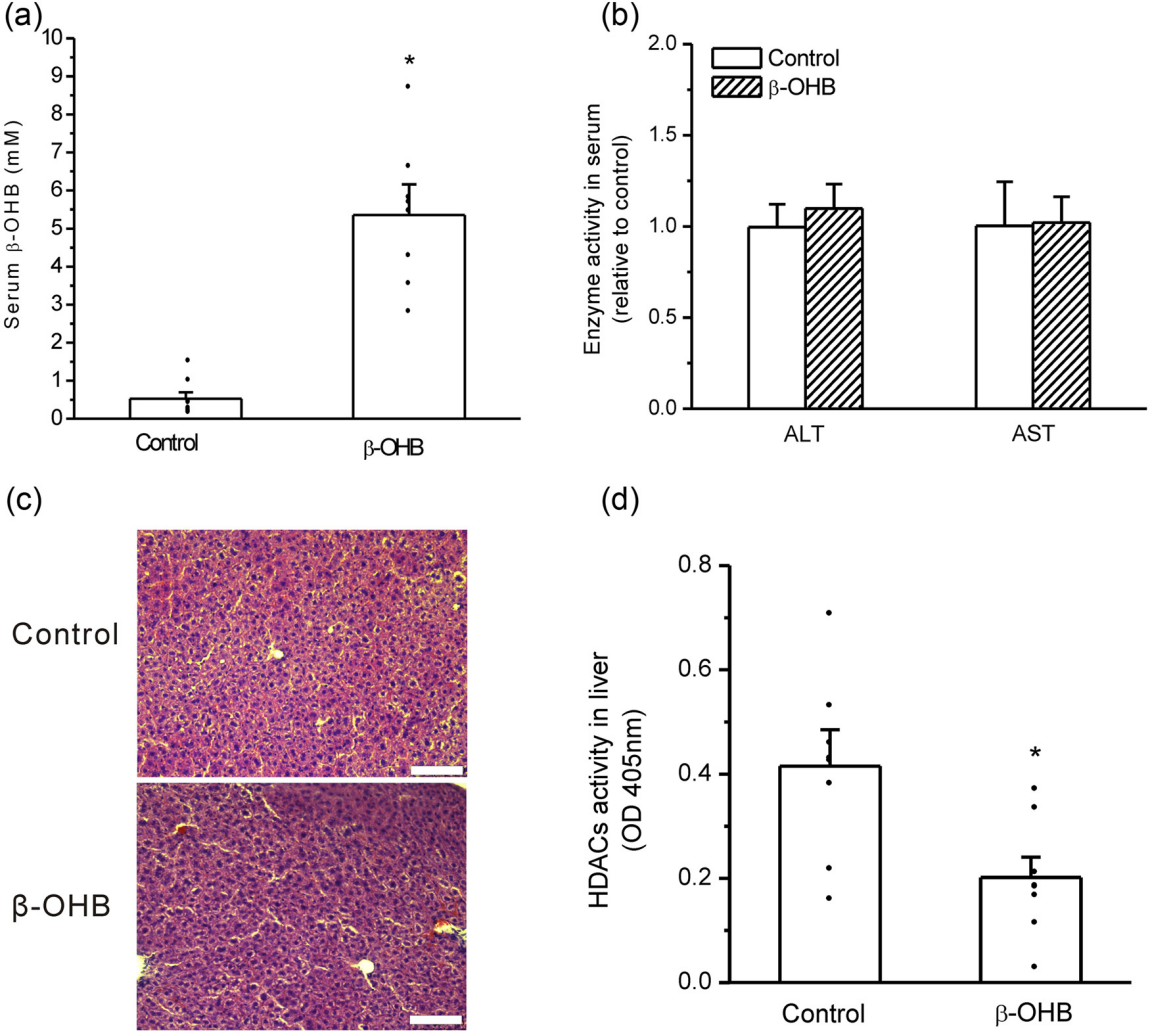
Acute increase in serum β-OHB inhibited HDACs’ activity in mouse liver. (a) β-OHB injections resulted in significant elevation of serum β-OHB (*P < 0.05 vs control, *n* = 8 mice per group); (b) β-OHB injections had no significant influence on ALT and AST activities in mouse serum (no significance vs control, *n* = 8 mice per group); (c) β-OHB injections had no significant influence on hepatic tissue structure (bar means 100 μm); and (d) β-OHB injections significantly inhibited HDACs’ activity in mouse liver (*P < 0.05 vs control, *n* = 8 mice per group).

**Figure 5:**
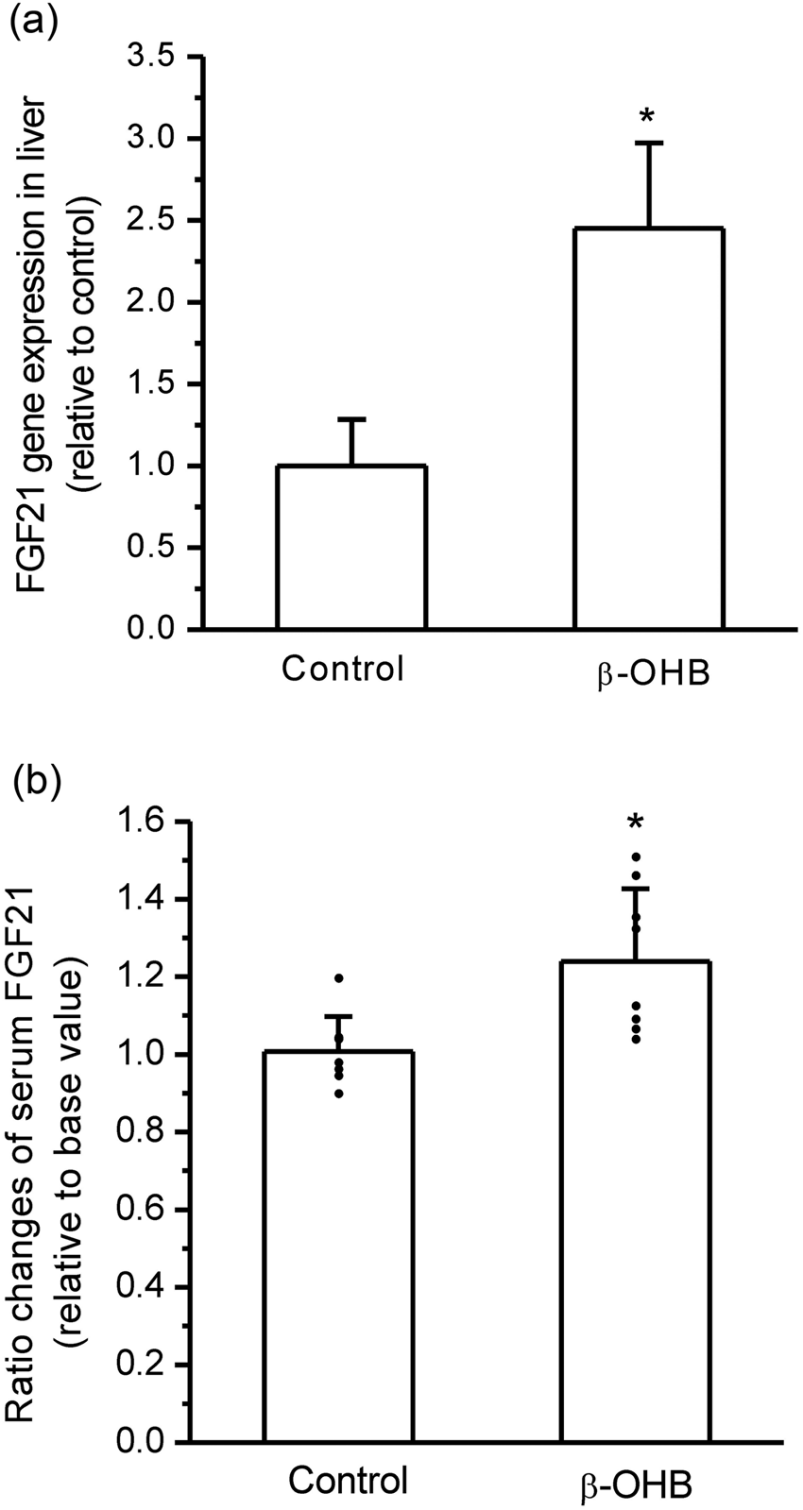
β-OHB stimulated FGF21 expression and secretion in mice *in vivo*. (a) β-OHB injections significantly upregulated FGF21 gene expression in mouse liver (*P < 0.05 vs control, *n* = 8 mice per group). (b) β-OHB injections significantly increased serum FGF21 levels in mice (*P < 0.05 vs control, *n* = 8 mice per group).

Serum β-OHB levels in mice significantly increased after intraperitoneal injections of β-OHB compared with the control (*P* < 0.05, *n* = 8, Figure 4a). To determine whether the toxic effect of β-OHB exists, the ALT and AST activities in mouse serum were measured, and they were not significantly different between the two groups (Figure 4b). H&E staining showed that the liver tissues had normal morphology in mice of the β-OHB-treated group without an obvious difference from the control (Figure 4c). HDACs’ activity in liver tissues was significantly inhibited after β-OHB treatment compared with the placebo control (*P* < 0.05, *n* = 8, Figure 4d). The mice in the β-OHB group exhibited a significant increase in FGF21 gene expression in the liver compared with the placebo control (*P* < 0.05, *n* = 8, Figure 5a). The ratio change in serum FGF21 of each mouse before and after β-OHB or placebo treatment was calculated, and it was found that serum FGF21 levels had a significant increase in β-OHB-treated mice compared with the placebo control (*P* < 0.05, *n* = 8, Figure 5b).

## 4 Discussion

This study demonstrates that β-OHB, the main ketone body, increases FGF21 expression and secretion in human HepG2 cells *in vitro* and in mouse liver *in vivo*. Hepatic FGF21 is upregulated during prolonged starvation to enhance ketone bodies’ production and their utilization [25]. The present study suggests that a positive feedback loop of FGF21 expression exists during starvation when ketone bodies become the important energy source, which gives another aspect of the interaction between FGF21 and ketone bodies.

β-OHB stimulated FGF21 expression from 2 mM *in vitro* in HepG2 cells. Serum β-OHB can surge up from less than 0.1 mM to more than 4 mM during starvation or ketogenic diet treatment, and it can exceed 10 mM under extreme conditions [19,20]. Therefore, serum β-OHB during both starvation and ketogenic diet treatment can sufficiently reach the level to upregulate FGF21 expression *in vivo*. The intraperitoneal injections of β-OHB in this study, which increased serum β-OHB levels to about 5 mM in mice, significantly upregulated hepatic FGF21 expression and increased serum FGF21 protein levels. These results support that β-OHB is an endogenous stimulator of FGF21 expression during starvation.

Previous studies have shown the concordant changes in the blood β-OHB and FGF21 both in humans and in mice, supporting the regulatory effects of β-OHB on FGF21 expression [26,27]. Nevertheless, the effects of ketone bodies on FGF21 expression remain controversial. An investigation showed that a ketogenic diet significantly reduced blood FGF21 levels while it reduced body weight in obese human subjects [28]. Recently, a human subject study found that the increase in the blood β-OHB did not result in the elevation of circulating FGF21 levels [29]. In contrast, studies in mice showed that a ketogenic diet upregulated the hepatic FGF21 expression [30,31]. The present study in mice observed the stimulatory effect of β-OHB on hepatic FGF21 expression. The discrepancy in the reports may result from species differences. Species divergence of gene expression in the cortex has been demonstrated between humans and mice [32]. A recent report indicates that many genes and biological pathways exhibit species-specific regulation in human and mouse livers [33]. On the other hand, metabolic differences exist between humans and mice, and they show diverse responses to nutrient status. For instance, FGF21 is rapidly induced after 6 h of fasting in mice, but it is not upregulated until 7–10 days of starvation in human subjects [1,34]. This difference in metabolism and gene expression may be the reason for the discrepancy in β-OHB-regulated FGF21 expression.

Ketone bodies are not only energy fuels but also signaling metabolites with multiple physiological functions. For example, β-OHB can act as a ligand for G protein-coupled receptors, such as GPR41 and GPR109A [35]. In addition, β-OHB functions as an endogenous inhibitor of HDACs to regulate gene expression in many types of cells [23,36]. In accordance with the previous reports, the present study found that β-OHB inhibits HDACs’ activity both in HepG2 cells *in vitro* and in mouse liver *in vivo*. HDACs’ inhibition by entinostat stimulated FGF21 expression in the present study. Therefore, it is suggested that inhibition of HDACs’ activity is one way for β-OHB to upregulate FGF21 expression, even if it is not the only way for β-OHB. HDACs generally act as transcription repressors because of their ability to induce the local condensation of chromatin [16,37]. HDACs’ inhibition has been shown to stimulate the expression of the genes that are related to the induction of tumor cell death and repression of oxidative stress [14,23,38]. It was reported that HDAC3 inhibition by butyrate stimulated FGF21 expression in mouse liver [17]. The present study further suggests that HDACs regulate FGF21 expression. In addition, it demonstrates that β-OHB inhibits HDACs’ activity and upregulates hepatic FGF21 expression at the concentration that can be reached during starvation. It is suggested that β-OHB-induced HDACs’ inhibition may be one reason for starvation-induced FGF21 expression and secretion.

Human HDACs’ belong to a family that includes 18 members, and they are classified into four groups [14,39]. Class I HDACs, which include HDAC1, 2, 3, and 8, are the most abundant HDACs localized in the nucleus [40,41]. A previous study indicates that β-OHB inhibits class I HDACs’ activity more efficiently than the other HDACs [23,36]. Entinostat is a specific inhibitor of class I HDACs [42], and it stimulates FGF21 expression. Moreover, no superposition effect of β-OHB on FGF21 expression was observed based on entinostat. These results suggest that β-OHB inhibits class I HDACs to stimulate hepatic FGF21 expression. Furthermore, because at least HDACs 1–8 are expressed in hepatocytes [43,44], it is suggested that more than one member of HDACs take part in the stimulatory effect of β-OHB on hepatic FGF21 expression. Nevertheless, the specific molecular mechanisms of β-OHB-regulated FGF21 expression is deficient in the present study and remain to be elucidated in the future.

FGF21 has beneficial metabolic actions and improves metabolic disturbances by alleviating the inflammation in metabolic disturbances as well as mobilizing glucose and lipid metabolism [5,45,46,47,48]. β-OHB increases in starvation and ketogenic diet treatment, and a ketogenic diet is an effective approach to treat metabolic disorders, such as type 2 diabetes and obesity [49,50]. Thus, the present study suggests that the upregulation of hepatic FGF21 expression possibly is one way by which a ketogenic diet achieves health benefits.
